# Unraveling Anxiety in Adults With Congenital Heart Disease: Untrodden Paths∗

**DOI:** 10.1016/j.jacadv.2023.100450

**Published:** 2023-07-27

**Authors:** Liesbet Van Bulck, Philip Moons

**Affiliations:** a KU Leuven Department of Public Health and Primary Care, KU Leuven, Leuven, Belgium; b Research Foundation Flanders (FWO), Brussels, Belgium; c Institute of Health and Care Sciences, University of Gothenburg, Gothenburg, Sweden; d Department of Paediatrics and Child Health, University of Cape Town, Cape Town, South Africa

**Keywords:** anxiety, congenital, heart defects, illness identity, illness perception, mental Health

**Figure:**
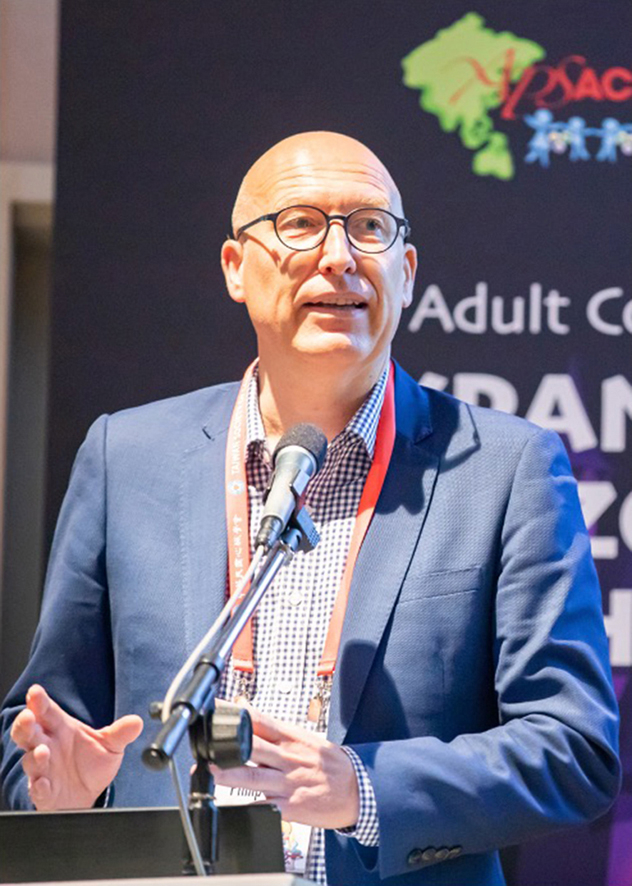

Living with congenital heart disease (CHD) poses unique and often complex medical and psychosocial challenges.^1^^,^^2^ The psychosocial challenges can be concerns about body image, adherence, sudden cardiac events, the evolution of their condition or mortality.^2^^,^^3^ Amidst this, anxiety can take root and intertwine with the challenges they face on a day-to-day basis. Indeed, anxiety is prevalent in this population. A recent meta-analysis in adults with CHD showed that 43% of patients reported at least mild anxiety symptoms, 22% reported moderate or severe anxiety symptoms, and 13% had a clinical diagnosis of an anxiety disorder.^4^

Although there is only sparse evidence on the impact of anxiety on health outcomes in CHD, literature suggests that high levels of anxiety increase the risk for adverse medical outcomes, premature mortality, and poorer quality of life.^5^, ^6^, ^7^ Hence, it should be a health priority to address anxiety symptoms in adults with CHD.^7^ To address anxiety in comprehensive, multidimensional, and tailored interventions, it is crucial to identify the (modifiable) predictors of anxiety.

Previous studies have identified several factors that predict higher levels of anxiety symptoms in adults with CHD.^2^^,^^8^, ^9^, ^10^ These predictors include being female, younger age, unemployment, higher NYHA functional class, feelings of loneliness, and fear of negative evaluation.^2^^,^^8^, ^9^, ^10^ Additionally, specific heart conditions, such as cyanotic heart disease/Eisenmenger syndrome or univentricular heart, have been associated with higher anxiety symptom scores.^1^^,^^11^ However, when accounting for functional status, the association between the heart defect and anxiety symptoms diminishes, indicating that functional status is a more significant factor in the development of anxiety symptoms than the anatomical complexity of the heart defect.^1^ Furthermore, other medical variables, including the functional status, have also been identified as predictors of anxiety in adults with CHD.^12^

While the medical and sociodemographic predictors of anxiety have received some attention, it is also crucial to acknowledge the role of other, more novel predictors in understanding and addressing anxiety in adults with CHD.^13^ Illness perceptions and illness identity are such novel predictors because there are indications that both constructs contribute significantly to the development and perpetuation of anxiety symptoms.^6^^,^^13^^,^^14^ However, the exact relationship between illness perception and anxiety symptoms has not yet been very well investigated in adults with CHD.^6^ Illness perceptions can be defined as the beliefs and subjective understandings that individuals hold about their illness, including their perceptions of its cause, consequences, timeline, controllability, and identity.^15^ Two studies have demonstrated an association between negative illness perceptions and increased anxiety levels in individuals with cardiovascular diseases.^13^^,^^16^ More specifically, lower scores on treatment control and coherence and higher scores on identity and emotional representation were associated with more anxiety symptoms in adults with CHD.^13^

In this issue of *JACC: Advances*, a paper from Marcil et al^17^ adds to the body of knowledge on illness experience and anxiety. The study aimed to examine the association between illness perception and anxiety symptoms in adults with CHD and the mediating role of coping in this relation. It was found that illness perception was associated with anxiety symptoms and that it even accounted for a greater proportion of variance than medical and sociodemographic variables combined. Also, they found that illness perception was associated with problem-focused coping, which was linked to reduced anxiety symptoms. The latter is an interesting new finding, as the combined effect of illness perceptions and coping on anxiety was not yet investigated in adults with CHD. These findings suggest that it could be worthwhile to focus on both illness perception and coping to intervene upon anxiety symptoms.^17^

Another concept of particular relevance in this respect is illness identity.^14^^,^^18^ Illness identity is the degree to which the chronic illness is integrated as part of the identity.^19^^,^^20^ As compared to illness perceptions, illness identity does not only expresses how a person views the illness but also how much the illness has impacted the way they think about themselves and the extent to which the illness is part of the sense of self.^20^ Illness identity encompasses 4 dimensions: engulfment, rejection, acceptance, and enrichment.^19^ Two papers have investigated the association between illness identity and anxiety in adults with CHD.^14^^,^^18^ These papers have shown that when adults with CHD felt overwhelmed by their disease, and thus did not integrate their disease well into their identity, they had higher levels of anxiety symptoms.^14^ Conversely, when patients accepted their disease as part of their identity, patients had lower levels of anxiety symptoms.^14^

The available literature indicates that anxiety in adults with CHD is not only linked to sociodemographic and medical variables but that also psychological factors such as illness perceptions and identity play a role.^13^ Health care providers of adults with CHD should be encouraged to walk the untrodden paths by assessing and recognizing illness perceptions and identity, and to adopt a patient-centered and holistic approach that acknowledges the emotional and psychological dimensions as well.^2^

There is a vital need to setup studies that can further deepen our understanding of the nature, prevalence, predictors, and their underlying pathways of anxiety in adults with CHD.^7^ To do so, longitudinal research is especially needed, which will allow to identify causal relationships and the direction of effect.^7^ While setting up interventions to improve mental health of adults with CHD, concepts such as illness perceptions and illness identity should be integrated.

## Funding support and author disclosures

This work was supported by the 10.13039/501100003130Research Foundation Flanders (FWO) (grant number 1159522N to Dr Van Bulck). The authors have reported that they have no relationships relevant to the contents of this paper to disclose.
